# The Efficiency of Antiosteoporosis Medicine after Intertrochanteric Fracture Surgery: A Retrospective Study of Refracture Rate, Function Recovery, Complications, and Mortality in the Chinese Elderly Population

**DOI:** 10.1055/s-0044-1779681

**Published:** 2024-02-12

**Authors:** Weidong Zhao, Shengbao Chen, Chao Tang, Changqing Zhang

**Affiliations:** 1 Department of Orthopaedics, General Hospital of Ningxia Medical University, Yinchuan, China; 2 Department of Orthopaedics, Shanghai Sixth People's Hospital, Shanghai Jiao Tong University, Shanghai, China

**Keywords:** elderly, intertrochanteric fracture, antiosteoporosis treatment, refracture

## Abstract

**Objective** This research aimed to discern the effects of antiosteoporosis medication on postoperative functional recovery, refracture incidence, complications, and mortality in geriatric patients with intertrochanteric fractures.

**Methods** A retrospective study was conducted on 250 patients aged 65 years and above who underwent surgery for intertrochanteric fractures between January 2013 and December 2014. Intertrochanteric fracture is diagnosed with International Classification of Diseases 10th Revision code (S72.101) and classified by the Evans–Jensen system. Collected data encompassed demographic details, pre- and postoperative histories of antiosteoporotic medication, functional outcomes (measured using Harris hip score, Parker Mobility Score, and EuroQol-5 Dimension [EQ-5D] scores), refracture incidences, complications, and survival rates. The antiosteoporotic regimen was categorized into essential (calcium, vitamin D) and advanced medications (bisphosphonate, calcitonin, etc.). Outcomes between patients on antiosteoporosis treatment (AO group) and those without (control group) were compared.

**Results** The cohort comprised 250 patients, with a gender distribution of 85 males (34%) and 165 females (66%), and a mean age of 79.8 ± 7.0 years. The median follow-up period was 15.82 months (maximum 31.13 months). Postoperatively, 126 (50.4%) patients were administered antiosteoporotic treatment. The refracture incidence in the AO group (2.4%, *n* = 3) was notably lower than the control group (8.9%, *n* = 11), manifesting a substantial risk reduction (odds ratio 0.251, 95% confidence interval 0.068–0.920, *p* = 0.024). While no marked differences in functional outcomes between the AO and control groups were observed (Harris score [96.17 ± 7.77 vs. 97.29 ± 6.74, *p* = 0.074), Parker score [8.54 ± 1.26 vs. 8.62 ± 1.18, *p* = 0.411], EQ-5D [0.83 ± 0.05 vs. 0.82 ± 0.06, *p* = 0.186]), patients administered a combination of essential and advanced drugs showcased significantly improved Harris and EQ-5D scores compared to those on essential drugs alone (Harris score [77.93 ± 2.04 vs. 84.94 ± 2.73, *p* = 0.015], EQ-5D [0.65 ± 0.03 vs. 0.75 ± 0.04, *p* = 0.015]).

**Conclusion** Postoperative antiosteoporosis treatment acts as a deterrent against refracture following intertrochanteric fracture surgeries, evidenced by a decline in refracture rates. However, the treatment's impact on functional recovery, quality of life, complications, and mortality remains indistinct. Interestingly, the combined administration of essential and advanced antiosteoporotic drugs seems to foster enhanced functional outcomes, warranting further exploration in future studies.

Hip fractures represent a paramount health concern globally, with a pronounced link to increased morbidity and mortality in the elderly. 1 Annually, over 1.7 million individuals sustain hip fractures, and a staggering 95% of these cases involve senior citizens. 2 With an ever-aging global populace, the annual incidence of elderly patients with hip fractures is projected to rise by 1 to 3%. Notably, half of these fractures are intertrochanteric. 2 The escalating occurrence of such injuries can be attributed to the combined effects of falls, muscular debility, and diminished levels of physical activity. 2 Additionally, the presence of osteoporosis further amplifies the risk of bone fractures. 3 Mortality after hip fracture is high (8–10%) within the first 30 days and around 20 to 28% in the first year, with approximately 30% directly attributable to the fracture itself. 4 Prior study suggested that active prevention of these complications can improve quality of life and reduce disease burden. 5 Continuing care for elderly hip fracture patients significantly improves quality of life, reduced anxiety, depression, and complications. 6 Currently, the primary therapeutic interventions for these fractures are internal fixation and arthroplasty.

While a plethora of research endeavors has sought to identify the determinants of clinical outcomes and the potential risks associated with surgical interventions for hip fractures, a core set of recommendations has emerged. 7 8 These encompass early surgical intervention, effective pain management during the perioperative phase, prophylaxis against thromboembolism and infections, prompt mobilization postsurgery, and measures to prevent falls. 9 Collectively, these strategies aim to bolster functional recovery while minimizing complications and mortality. 10 The efficacy of antiosteoporosis treatments for osteoporotic patients is well established. Various studies have suggested that osteoporosis treatment after hip fracture resulted in meaningful reductions in subsequent fractures and has a potential protective effect on the 5-year mortality rate. 9 11 12 Yet, there remains a knowledge gap regarding the influence of antiosteoporosis medications on clinical and functional outcomes following hip fracture surgeries, particularly within the elderly Chinese demographic. 13

Given this backdrop, the aim of our retrospective study was to elucidate the effects of antiosteoporosis medications on postoperative functional recovery, refracture incidence, complications, and mortality, utilizing data from our geriatric cohort with intertrochanteric fractures who underwent surgical treatment.

## Patients and Methods

### Patient Data Sources

**Fig. 1:**
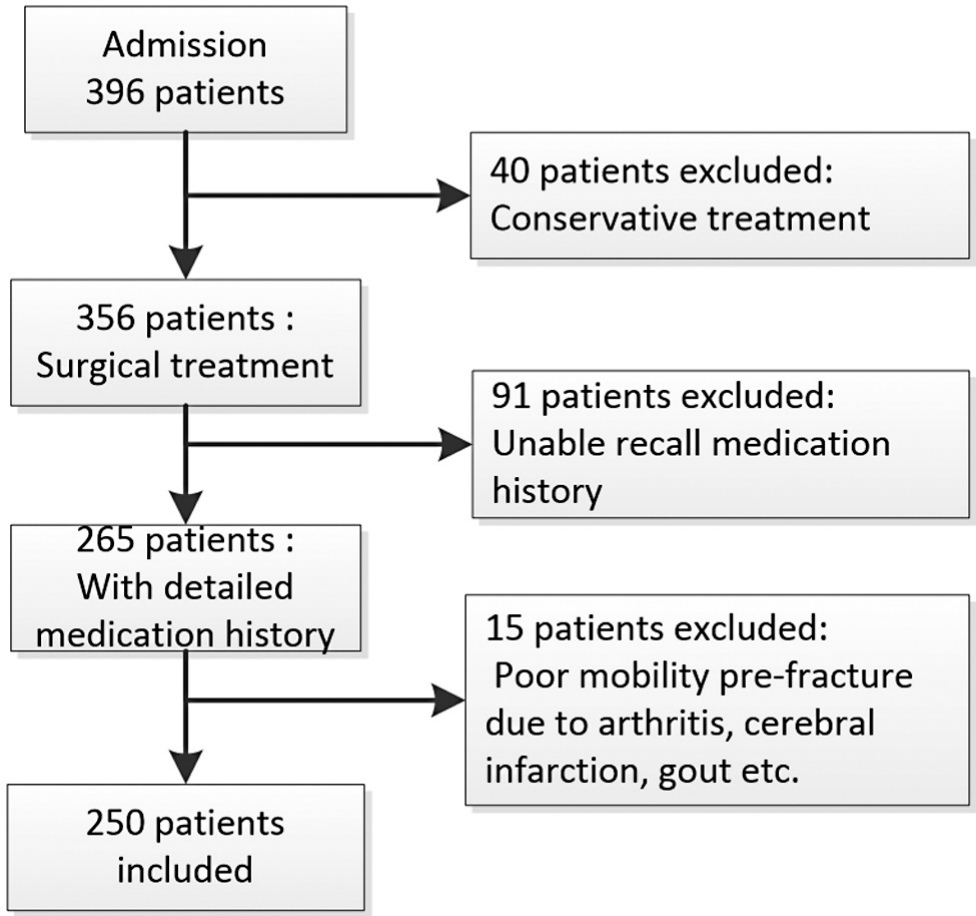
Flowchart for patient selection.

We sourced the records of geriatric patients with intertrochanteric fractures from January 2013 to December 2014 from the orthopaedic department database of our hospital. Inclusion criteria entailed patients who were ( Fig. 1 ):

1. Sixty-five years or older (regardless of gender),
2. Diagnosed with closed intertrochanteric hip fractures confirmed by X-ray (International Classification of Diseases 10th Revision S72.101),
3. Treated with surgical internal fixation or arthroplasty, and
4. Able to walk with or without aids indoor/outdoor.

Patients were excluded if they had:

1. Pathological fractures due to tumors or other conditions,
2. Infected wounds at the fracture site,
3. Undergone conservative treatment,
4. Fractures older than 3 weeks, and
5. An inability to recall their osteoporosis medication history.

Our study received the requisite approval from our hospital's Regional Ethics Committee, and informed consent was obtained from all participating patients.

Data parameters for our study encompassed: patient identity, demographic details, fracture specifics, pre- and postoperative antiosteoporotic medication history, functional assessment (both pre- and postoperation) using the Harris hip score (Harris score), 14 Parker Mobility Score (Parker score), 15 and EuroQol-5 Dimension (EQ-5D) score, 16 perioperative treatment details, refracture incidences, complications, and survival. For the EQ-5D utility score, we utilized the utility scale table from Japan, given the absence of a suitable scale for the Chinese population, citing the close ethnic and geographical ties between China and Japan. 16

Of the 396 patients with complete data, 146 were excluded for various reasons: 40 underwent conservative treatment, 91 could not recall their osteoporosis medication history, and 15 had compromised mobility before the fracture due to various conditions. This left 250 patients for the study.

### Data Analysis Method

Our cohort was bifurcated into two segments:

1. Patients receiving antiosteoporosis treatment (AO group) and
2. Those not on any such treatment (control or CO group).

For inclusion in the AO group, patients must have consistently taken antiosteoporotic medications for a minimum of 3 months postsurgery. This group was further segmented based on medication type: essential (A group), advanced (B group), both essential and advanced (C group), and unclear categorization (D group, comprising deceased patients or those unable to recall medication specifics). Essential medications included calcium and vitamin D (or activated vitamin D), while advanced encompassed bisphosphonate, calcitonin, parathyroid hormone (PTH), and Xianlinggubao (a traditional Chinese medicine).

Injury etiologies categorized fractures as either low-energy (resulting from standing height falls or without clear causes) or high-energy (from vehicular accidents, high falls, or significant collisions).

Furthermore, cardiovascular conditions included hypertension, coronary heart disease, and atrial fibrillation, while neurological ailments covered Alzheimer's, cerebral infarction, and Parkinson's. A fall history denoted any fall in the preceding year. Early complications were defined as those arising within 3 months postsurgery, and late complications as those manifesting after this period. Surgical techniques encompassed InterTAN (Smith & Nephew company), Gamma3 (Stryker Corporation), locking plate (Johnson & Johnson), and total hip arthroplasty (LINK).

Postoperative complications were also categorized: infections included wound, deep site, pulmonary, and urinary tract infections; instrument-related complications covered issues like screw loosening, nail withdrawal, limb shortening, etc.

### Statistical Analysis

All collated data were cataloged in Excel and analyzed using SPSS (version 22.0; Chicago, IL). Enumerative data were represented as frequencies. For normally distributed measurement data, we used the mean ± standard deviation; for nonnormally distributed data, the median range (lower quartile to upper quartile) was adopted. Data adhering to a normal distribution were analyzed using the *t* -test or one-way analysis of variance, while the Kruskal–Wallis test was employed for nonnormally distributed data. The Pearson's chi-square test was used for enumerative data, with the Fisher's exact test deployed for smaller sample sizes. Covariance analysis was applied for functional recovery comparisons (Harris score, Parker score, and EQ-5D score). A *p* -value of < 0.05 was deemed statistically significant.

## Results

The demographic and clinical characteristics of the patients are summarized in Table 1 .

**Table 1:** Baseline information of study cohort

Item	Total	AO group	CO group	*p* -Value
Cases ( *n* )	250	126	124	
Age (mean ± SD)	79.77 ± 7.04	79.71 ± 7.20	79.84 ± 6.88	0.872
Gender (male/female)	85/165	39/87	46/78	0.186
Fracture side (left/right)	130/120	59/67	61/63	0.402
Evans–Jensen type				
Ia	12	6	6	0.731
Ib	37	17	20	
IIa	20	10	10	
IIb	96	45	51	
III	85	48	37	
Hospital stay (d)	8.77 ± 3.6	8.14 ± 2.97	9.35 ± 4.05	0.001
Medical history				
Diabetes	43	23	20	0.656
Cardiovascular system disease	94	51	43	0.344
Chronic respiratory disease	28	13	15	0.656
Nervous system diseases	41	20	21	0.821
Osteoporosis	107	61	46	0.071
History of fracture	25	19	6	0.007
History of hip fracture	7	3	4	0.686
History of fall	6	5	1	0.102
Nonviolent fracture	4	3	1	0.321
Multifracture (this time)	13	8	5	0.409
Trauma energy (low/high)	213/37	108/18	103/21	0.564
Preoperative waiting time				
< 48 h	18	7	11	0.435
48–72 h	57	29	28	
72–96 h	62	36	26	
> 96 h	111	53	58	
Preoperative Harris score	96.72 ± 7.3	96.17 ± 7.77	97.29 ± 6.74	0.074
Preoperative Parker score	8.58 ± 1.22	8.54 ± 1.26	8.62 ± 1.18	0.411
Preoperative EQ-5D	0.83 ± 0.05	0.83 ± 0.05	0.82 ± 0.06	0.186
Preoperative hemoglobin (g/L)	106.0 ± 17.0	106.1 ± 16.9	105.9 ± 17.8	0.793
Preoperative albumin (g/L)	36.8 ± 4.8	37.0 ± 4.7	36.7 ± 4.9	0.266
Surgery methods				
InterTAN	122	52	71	0.041
Gamma3	82	45	36	
Locking plate	41	27	14	
Total hip arthroplasty	5	2	3	
Preoperative antiosteoporotic drugs				
Essential drugs	30	24	6	0.001
Advanced drugs	4	3	1	0.321

Abbreviations: AO, antiosteoporosis treatment group; CO, control group; EQ-5D, EuroQol-5 Dimension; SD, standard deviation.

Our analysis encompassed data from 250 patients, consisting of 85 males (34%) and 165 females (66%). The average age of the participants was 79.8 ± 7.0 years, with a follow-up period averaging 15.3 ± 8.2 months. A notable 50.4% ( *n* = 126) of the patients with intertrochanteric fractures were found to be on some form of antiosteoporotic medication (AO group). This indicates that the remaining 49.6% ( *n* = 124) did not receive any such treatment postfracture.

Diving deeper into the data, 15.1% (19/126) of the AO group had experienced a fracture prior to the current incident, compared to a smaller 4.8% (6/124) in the CO group ( *p* < 0.05). When considering the use of essential antiosteoporotic drugs specifically, 19% (24/126) of the AO group were on these medications, in contrast to 4.8% (6/124) in the CO group ( *p* < 0.05).

In terms of the causative factors behind the fractures, a significant 85.2% (213/250) were attributed to low-energy incidents, while the remaining 14.8% (37/250) resulted from high-energy events.

Within the AO group, calcium and vitamin D emerged as the most commonly prescribed antiosteoporosis medications, accounting for 42.1% ( *n* = 53) of the treatments. Calcitonin was administered to 30.9% ( *n* = 39) of patients, Xianlinggubao to 9.5% ( *n* = 12), bisphosphonate to 7.1% ( *n* = 9), and PTH to a mere 2.3% ( *n* = 3). A closer look reveals that approximately 48.4% of the patients were on a singular medication, while 22.2% were prescribed a combination of two different drugs.

Turning our attention to refracture instances, 14 patients experienced this complication. When dissecting the data, the refracture rate in the AO group stood at 2.4% ( *n* = 3), which was notably lower than the 8.9% ( *n* = 11) observed in the CO group. This statistical difference translated to reduced odds (odds ratio [OR] 0.251, 95% confidence interval [CI] 0.068–0.920, *p* = 0.024), as detailed in Table 2 . Intriguingly, all 14 refracture cases were females, making their risk of experiencing a refracture 1.56 times higher than their male counterparts (95% CI 1.42–1.72, *p* = 0.002).

**Table 2:** Refracture characteristics of AO group and CO group

Item	AO group ( *n* = 126)	CO group ( *n* = 124)
Refracture ( *n* )	3 a	11 a
Gender (male/female)	0/3 b	0/11 b
Refracture free time (d)	171 ± 128	222 ± 169
Refracture site		
Hip fracture	1 c	7 c
Other site	2	5
Refracture vs. weight bearing		
Before first weight-bearing	2	2
Partial weight-bearing	0	2
After full weight-bearing	1	6
Refracture with fall (yes/no)	2/1	6/3
Death	0	2

Abbreviations: AO, antiosteoporosis treatment group; CI, confidence interval; CO, control group; OR, odds ratio.

a OR = 0.251, 95% CI 0.068–0.92, *p* = 0.024.

b OR = 1.563, 95% CI 1.42–1.72, *p* = 0.002.

c OR = 0.134, 95% CI 0.016–1.103, *p* = 0.031.

Of these 14 refracture cases, 8 were hip fractures. Within this subset, the AO group had a 0.8% ( *n* = 1) incidence rate, in contrast to the 5.6% ( *n* = 7) recorded in the control group ( *p* < 0.05). A trend was identified among the CO group patients with refractures: they were more inclined to experience a hip fracture post their full weight-bearing time. The specifics regarding the refracture characteristics for both the AO and CO groups are comprehensively detailed in Table 2 .

In the duration of our study, a mere 16% (40 out of 250) of the patients underwent bone mineral density (BMD) assessments postsurgery, owing to various reasons. From this subset, 57.5% (23 patients) had been administered antiosteoporosis treatment, while the remaining 42.5% (17 patients) had not received any. Upon comparing the two factions, the adjusted BMD values for L1-4 spine, femoral neck, and total hip did not exhibit any significant disparities: 0.904 ± 0.042 versus 0.879 ± 0.050 ( *p* > 0.05), 0.690 ± 0.024 versus 0.635 ± 0.028 ( *p* > 0.05), and 0.740 ± 0.025 versus 0.696 ± 0.029 ( *p* > 0.05), respectively. However, it is worth noting that due to the limited number of patients having prefracture BMD evaluations, drawing a definitive correlation between postsurgical BMD and antiosteoporosis treatment remained elusive.

**Fig. 2:**
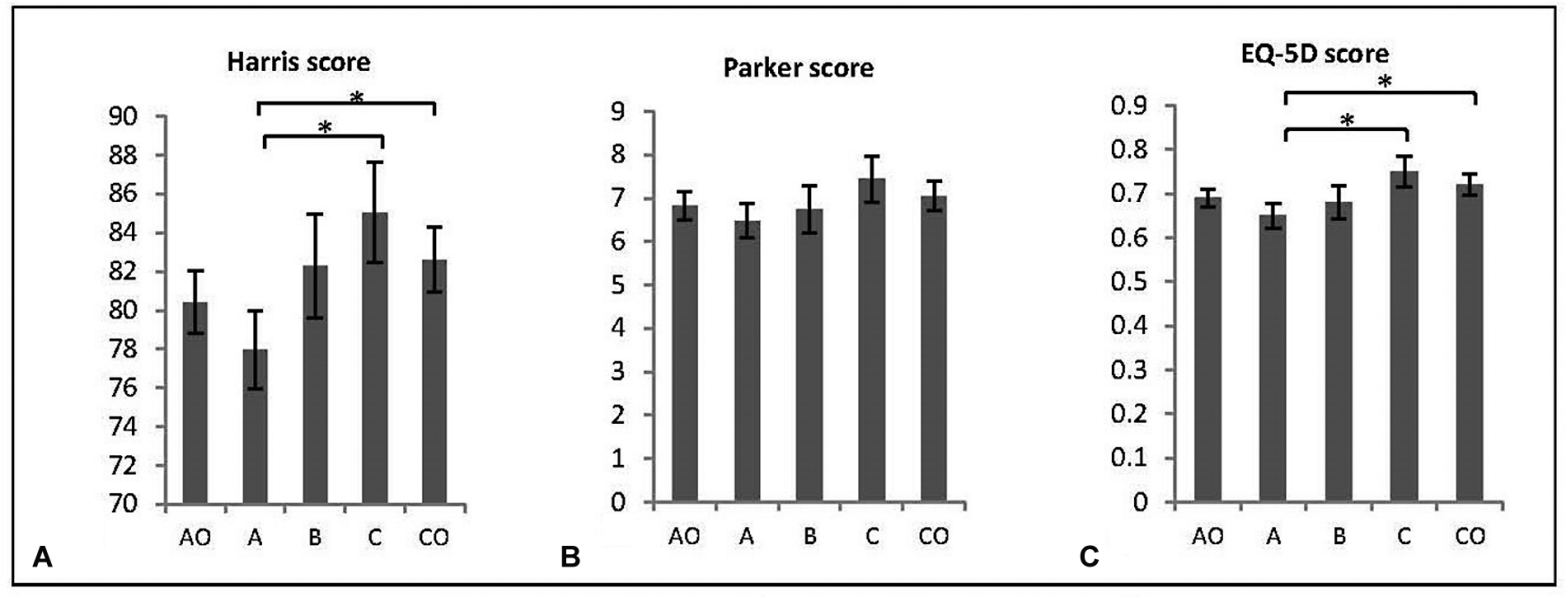
Postoperative functional outcome. ^a^ ( **A** ) Harris score: group A vs. CO group ( *p* = 0.013); group A vs. group C ( *p* = 0.015). ( **B** ) Parker score: no significant difference between group A and CO group, and group A and group C. ( **C** ) EuroQol-5 Dimension (EQ-5D) score: group A vs. CO group ( *p* = 0.008); group A vs. group C ( *p* = 0.015). ^a^ Postoperative functional outcome adjusted by covariate analysis. Because information of antiosteoporosis drugs of 28 patients in group D was unknown, comparisons were made between other groups. AO group: antiosteoporosis treatment; group A: essential medication; group B: advanced medication; group C: essential and advanced medication; CO group: control.

Further analysis revealed that 50.4% ( *n* = 126) of the hip fracture patients were on some form of antiosteoporotic medication (AO group). Comparative evaluations of the Harris scores, Parker scores, and EQ-5D scores between the AO and control groups did not yield any significant differences. When we further categorized patients based on their specific antiosteoporosis medications, the distribution for groups A, B, C, and D stood at 38.9% (49 patients), 17.5% (22 patients), 21.4% (27 patients), and 22.2% (28 patients), respectively. Owing to the uncertain medication data for group D, our comparisons were restricted to groups A, B, and C. Patients who were on a combination of essential and advanced antiosteoporosis medications, as well as those not on any treatment, demonstrated significantly elevated Harris and EQ-5D scores when compared against those solely on essential medications. This comparison is visually represented in Fig. 2 . Factors influencing postoperative functional recovery, such as age, presurgery functional scores, and the timing of the first weight-bearing postsurgery, are detailed in Table 3 .

**Table 3:** Covariate analysis of postoperative Functional outcome a

Source	Harris score		Parker score		EQ-5D	
	*F*	Sig.	*F*	Sig.	*F*	Sig.
Corrected model	3.610	0.000	4.446	0.000	2.734	0.000
Intercept	16.633	0.000	23.400	0.000	1.886	0.171
Age (y)	12.834	0.000	30.857	0.000	5.857	0.016
First weight-bearing (mo)	8.927	0.003	2.570	0.111	10.188	0.002
Preoperative Harris score	24.849	0.000	26.769	0.000	17.847	0.000
Surgery method	0.565	0.688	1.464	0.215	0.557	0.694
Antiosteoporosis drug group	2.512	0.043	1.189	0.317	2.523	0.043
Surgery method*antiosteoporosis drug group	.0765	0.685	.524	0.897	0.848	0.601

Abbreviations: EQ-5D, EuroQol-5 Dimension; Sig., significance.

a Covariate analysis of postoperative functional outcomes among group A (essential medication), group B (advanced medication), group C (essential and advanced group), and CO group (control).

When investigating early complications, most did not exhibit a discernible correlation with osteoporotic treatment. A notable exception was the incidence rate of deep venous thrombosis in the AO group, which stood at 27.3% (6/22)—markedly higher than the 9.7% (12/124) observed in the control group.

Regarding the postsurgical full weight-bearing duration, the AO group and control group did not differ significantly. However, a more granular examination revealed that the subgroup prescribed a combination of essential and advanced antiosteoporosis treatments showcased a prolonged full weight-bearing time (152.73 ± 64.06) postsurgery. This was in contrast to the essential drugs-only group (group A) which averaged 125.23 ± 48.83, the advanced drugs-only group (group B) which averaged 107.86 ± 49.34, and the control group which averaged 124.67 ± 53.27. These distinctions are meticulously detailed in Table 4 .

**Table 4:** Postoperative complication and mortality

Item	In total	AO group					CO group
		Sum	Group A	Group B	Group C	Group D	
Cases ( *n* )	250	126	49	22	27	28	124
First weight-bearing (d)	75.9 ± 41.9	76.0 ± 39.6	81.2 ± 37.0	60.3 ± 35.2	86.6 ± 41.4	68.9 ± 41.3	75.9 ± 44.1
Full weight-bearing (d)	125.1 ± 53.9	125.6 ± 54.5	125.2 ± 48.8 a	107.9 ± 49.3 a	152.7 ± 64.1 a	115.7 ± 48.1 a	124.7 ± 53.3 a
Early complication							
DVT	32	20	7	6 b	4	3	12 b
Infection	4	2	0	0	1	1	2
Pressure sore	2	1	0	1	0	0	1
Wound pain	2	1	0	1	0	0	1
Other c	4	1	0	0	0	1	3
Late complication							
Consistent pain	11	5	5	0	0	0	6
Restricted joint movement/stiffness	40	19	11	0	0	3	21
DVT	27	18	7	3	4	4	9
Instrument-related complications	13	7	6	2	0	0	5
Malunion	3	1	0	0	0	1	2
Other d	6	3	2	1		0	3
Death	15	7	1	0	0	6	8

Abbreviations: AO, antiosteoporosis treatment group; CO, control group; DVT, deep vein thrombosis.

a Group C vs. group A, group B, group D, group D, *p* < 0.05.

b Group B vs. CO group, *p* < 0.05.

c Cerebral infarction, incontinence, constipation, and implant rejection.

d Infection, muscle atrophy, pressure sore, and paresthesia.

Lastly, the overall mortality rate postsurgery, which stood at 6% (15/250), did not seem to be significantly influenced by the administration of antiosteoporosis treatments. The AO group recorded a mortality rate of 5.5% (7/126), whereas the control group was slightly higher at 6.45% (8/124), as further delineated in Table 4 .

## Discussion

Osteoporosis, a condition characterized by weakened bone strength and a consequent heightened susceptibility to fractures, is driven by two primary factors: bone quality and BMD. Disturbingly, osteoporosis remains a silent affliction for many; its presence often only becomes palpable following a fragility fracture. The prevalence of osteoporosis is staggeringly high in the elderly demographic, while the benefits of antiosteoporosis treatments for patients diagnosed with osteoporosis are unequivocally acknowledged. This underscores the urgency to fathom the interplay between osteoporotic medications and fracture healing, to fine-tune both osteoporosis management and fracture treatments. 17

Our study underscored the overwhelming prevalence of fragility fractures among the elderly suffering from intertrochanteric fractures. Recent research from the U.K., encompassing a broad sample of over 27,542 hip fracture patients aged 50 and above, indicated a rising trend of post-hip fracture osteoporosis medication prescriptions, soaring from a mere 7% in 2000 to an impressive 46% by 2010. 18 This shift was even more pronounced among patients aged 75 and above. 18 Another expansive study spotlighted factors like age, prior hip fractures, and corticosteroid usage as key drivers amplifying the likelihood of osteoporosis medication prescriptions. 19 Yet, dementia, obesity, and exposure to opioid analgesics or psychotropic drugs seemed to temper this enthusiasm. 18 With osteoporosis prevalence pegged at 42.8% (48.4% for the AO group and 37.1% for the CO group, *p* = 0.073), postoperative osteoporosis medication consumption was recorded at 50.4%. Our data further illuminated the prevailing preferences: calcium and vitamin D emerged as the go-to essential osteoporosis treatments (42.1%), while calcitonin (30.9%) and bisphosphonates (7.1%) dominated the advanced treatment landscape. Remarkably, nearly half (48.4%) of the patients relied on just one medication, and a little over a fifth (22.2%) opted for a dual-drug regimen. This underscores a growing awareness and emphasis, both by patients and health care professionals, on the pivotal role of osteoporosis medications in postfracture recovery.

Within the demographic of hip fracture patients, the incidence of a second hip fracture ranges from 2 to 10%. 20 An individual who has sustained one hip fracture is 3 to 9 times more susceptible to a subsequent one. 18 21 22 While the risk of refracture peaks within the first year post the initial fracture, it remains considerably high in the following 5 years. 18 The potential of antiosteoporosis treatment in mitigating the risk of refracture is evaluated. Animal experiments have elucidated the crucial role antiosteoporosis drugs play in the fracture healing process. Bisphosphonates, for instance, while delaying the maturation rate of callus following cartilage calcification, also amplify callus volume, BMD, and bolster mechanical properties. 18 Larsson and Fazzalari corroborated these findings, emphasizing that bisphosphonates, while decelerating remodeling, did not exhibit any evidence of hindering healing. 23 Other drugs like calcitonin have been identified to promote early endochondral ossification and enhance torsional strength and stiffness. 24 Similarly, PTH augments early endochondral repair, thereby escalating callus volume, density, and maturity. Traditional Chinese medicine, Xianlinggubao, has also been highlighted to increase callus volume, BMD, and trabecular bone, while accelerating endochondral ossification. 25 26 In our investigation, 14 out of 250 patients (5.6%) experienced a refracture. When juxtaposed against the control group's 8.9% (11/124) refracture rate, the antiosteoporosis group (2.4%, 3/126) exhibited a notably reduced refracture risk (OR 0.251, 95% CI 0.068–0.921). A more granular analysis revealed that the hip refracture rate within the AO group (0.8%) also undercut the CO group's 5.6% rate. The limited number of refracture incidents precluded a nuanced analysis based on drug categories. Yet, the prevailing research consensus underscores that consistent and compliant utilization of antiosteoporosis drugs, notably bisphosphonates, can curtail refracture risks. 27 Hegde et al articulated that bisphosphonates can diminish vertebral and hip fracture risks by a staggering 70 and 50%, respectively. 17 A multifaceted analysis by Shen et al spotlighted bisphosphonates as a bulwark against refractures. They further identified a suite of refracture risk factors post-hip fracture surgery, which included age, obesity, female gender, and a history of diabetes, among others. 28 Our study echoed these findings, especially underscoring the elevated refracture risk among women.

Postoperative functional recovery, representing a restoration to preinjury mobility and overall quality of life, remains the paramount concern for patients. Instruments like the Harris score, Parker score, and EQ-5D scores are pivotal in gauging functional recovery, mobility, and the overall quality of life in intertrochanteric fracture patients. Seng et al's findings suggest that vitamin D deficiency, though crucial in many physiological processes, did not significantly hamper functional recovery post-hip fracture surgery. 29 A vital component of the postoperative functional recovery metric is pain management, a predominant criterion in both Harris and EQ-5D evaluations. 14 16 Knopp-Sihota et al's meta-analysis highlighted calcitonin's efficacy in mitigating acute pain, especially within the first month postsurgery, but its efficacy waned in managing chronic pain. 30 Furthermore, Huusko et al's study indicated that while intranasal calcitonin might expedite recovery to some extent, its role in pain management remained inconclusive. 31 However, more recent studies suggest a correlation between the administration of antiosteoporosis drugs, like bisphosphonates, and better postoperative functional recovery. 13 Our research revealed that while there was no discernible difference in postoperative Harris, Parker, and EQ-5D scores between the AO and CO groups, a nuanced observation indicated a superior Harris and EQ-5D score in patients who were administered a combination of essential and advanced antiosteoporosis medications compared to those who only received essential drugs. This infers that advanced antiosteoporotic drugs, when complemented with essential ones, can usher in enhanced functional outcomes.

Postoperative complications following intertrochanteric fractures encompass a myriad of challenges, with deep vein thrombosis (DVT), infections, restricted joint mobility, joint stiffness, and hardware-related complications being particularly prevalent, especially after hospital discharge. 32 33 In our analysis, we observed that restricted joint movement or stiffness was a significant complication, with its occurrence noted in 16% (40/250) of patients. Interestingly, many patients associated this complication with a deficiency in postoperative rehabilitation exercises. On dissecting various early and late complications, including infections, persistent pain, limited joint mobility, and device-related complications, no significant disparities were evident between the AO and CO groups. A salient observation, however, was the elevated incidence of DVT in the AO group (27.3%, 6/22) compared to the CO group (9.7%, 12/124). Further scrutiny, focusing on preoperative lower limb vessel ultrasonography data, helped in isolating patients already predisposed to DVT. Upon excluding these patients, the differential in DVT rates between the two groups was rendered insignificant (18.2%, 4/22 for the AO group vs. 4.8%, 6/124 for the CO group). Our study suggests that antiosteoporosis medications, while vital for bone health, exert a minimal influence on postoperative complications. As such, more comprehensive strategies encompassing patient health assessment and rigorous postoperative rehabilitation are imperative to minimize these complications.

Intertrochanteric fractures predominantly afflict an older demographic, commonly entwined with other chronic health conditions. 34 As per existing literature, hip fractures precipitate an uptick in mortality rates, with figures hovering around 10% at 30 days postfracture, escalating to a range of 15 to 25% by the 1-year mark. 34 35 This amplified mortality postfracture is multifactorial, with age, gender, the magnitude of complications, cognitive state, anemia, and subpar living conditions serving as key contributory elements. 34 In our cohort, the postoperative mortality rate stood at 6%. Intriguingly, the mortality figures for patients under antiosteoporosis treatment (5.5%) did not showcase any statistical deviation when juxtaposed with their counterparts not under such treatment (6.45%). Brozek et al, in their expansive study encompassing 31,668 hip fracture patients aged 50 and above, propounded that commencing bisphosphonate postfracture curtailed mortality risks, with females in particular witnessing a substantial 57% plunge in mortality rates. 1 Parallelly, Lyles et al illuminated, through a randomized double-blind placebo-controlled trial with a sample size of 2,111 hip fracture patients aged 50 or more, that patients administered zoledronic acid postsurgery registered a comparatively lower mortality rate of 9.6% (101 out of 1,054) vis-à-vis the placebo group that had a rate of 13.3% (141 out of 1,057). This translates to a 28% decrement in mortality under the aegis of zoledronic acid. 35 Huusko et al, drawing from a randomized double-blind study of 260 hip fracture patients aged 65 and above, posited that calcitonin did not wield any discernible influence on postoperative mortality stemming from hip fractures. 31 Similarly, Makridis et al's longitudinal study monitoring 520 postoperative hip fracture patients found the mortality impact of antiosteoporotic medications (predominantly bisphosphonates) to be negligible. 13 In synthesizing these findings, one could postulate that the mortality-modulating prowess of antiosteoporosis medications might be contingent on the specific drug in question. Our study, constrained by its sample size, could not delve into a nuanced mortality analysis stratified by the specific antiosteoporosis medication categories.

Intertrochanteric fractures are challenging scenarios, especially in the geriatric population. Our study sheds light on the pivotal role antiosteoporosis treatment plays postsurgery, specifically in diminishing the likelihood of refractures. However, when we consider other critical parameters like functional recuperation, life quality, complications, and mortality rates, the impact of antiosteoporosis treatment appears more nuanced. In essence, while the immediate benefits of antiosteoporosis treatment in reducing refracture rates are evident, its broader implications in the postoperative journey of intertrochanteric fracture patients warrant deeper exploration.
